# Supplementary material for: Low-Diversity Microbiota in Apical Periodontitis and High Blood Pressure Are Signatures of the Severity of Apical Lesions in Humans
Source: Int J Mol Sci. 2023 Jan 13;24(2):1589. doi: 10.3390/ijms24021589 (PMC9866854; doi:10.3390/ijms24021589)
Supplement: Supplementary file 1 [file ijms-24-01589-s001.zip › ijms-2132546-supplementary.pdf]

	**Low diversity (n=43)**	**High diversity (n=51)**	**p value**
**Age (years)**	53.58 ± 12.72	55.09 ± 14.64	0.30
**Weight (kg)**	69.97 ± 12.69	72.35 ± 14.43	0.242
**Height (cm)**	171.16 ± 9.12	170.43 ± 7.167	0.301
**BMI (kg/m^2)**	23.73 ± 2.77	24.79 ± 4.06	0.083
**Stress Score (Scare 0 to 10)**	5.18 ± 2.58	4.35 ± 2.8	0.066
**DMF (Decayed, Missing, Filled)**	14.65 ± 4.99	14.88 ± 5.59	0.83
**Decayed (D)**	0.37 ± 0.90	0.60 ± 1.078	0.121
**Missing (M)**	9.13 ± 3.04	8.72 ± 4.59	0.184
**Filled (F)**	5.55 ± 3.34	5.74 ± 4.28	0.382
**Number of dental brushing/day**	1.93 ± 0.59	2.07 ± 0.59	0.23
**Score PAI**	3.95 ± 1.13	3.41 ±0.98	**0.01**
**Diagnostic of severe peri apical diseases (PAI =5)**	46.5 % (n=20)	15.6 % (n=8)	Odds Ratio : 4.592 IC 95% [1.6329 ; 14.0728]; **p=0.001**

Supplementary Table S1: Clinical and oral parameters based on the diversity score (data as mean ± SD. *p<0.05. **p<0.01. ****p<0.0001. unpaired Mann-Whitney test).

Relative abundance (%) in oral microbiota	Microbiota parameters among the High div (n=51)	PAI≤3	PAI >3	p value
	α-diversity by the Shannon index	1.96±0.83	1.57 ± 0.91	**0.0370**
Family	Burkholderiaceae	0.90±1.84	0.16±0.74	**0.0012**
	Flavobactereriaceae	1.03±4.48	0.34±1.34	**0.18**
	Pseudomonadaceae	8.40±21.49	10.18±22.21	**0.22**
Genus	Propionibacterium	0.00±0.00	0.41±0.92	**<0.0001**
	Prevotella7	1.39±5.02	2.14±4.64	**0.17**
	Capnocytophaga	0.84±3.95	0.33±1.34	**0.36**
	Butyrivibrio2	0.02±0.10	0.44±1.69	**<0.0001**
	Sphingomonas	0.87±1.63	0.44±1.69	**0.0348**
	Pseudomonas	8.40±21.49	10.18±22.21	**0.22**

Supplementary Table S2: Relative abundance (%) for taxonomic family and genus identified with significant differences in granuloma microbiota in the two groups among the high diversity samples. (data as mean ± SD. *p<0.05. **p<0.01. ****p<0.0001. unpaired Mann-Whitney test).

Relative abundance (%) in oral microbiota	Microbiota parameters among the High div (n=51)	HBP +	HBP -	p value
	α-diversity by the Shannon index	1.82 ± 0.93	1.45± 0.87	0.0434
	α-diversity by the Chao Index	92.66 ± 25.76	82.04 ± 19.07	0.045
Family	Actinomycetaceae	0.016±0.46	1.41±3.62	**0.0025**
	Corynebacteriaceae	0.02±0.07	0.54±1.65	**0.0035**
	Muribaculaceae	0.43±1.73	0.090.56	**0.0914**
	Sphingobacteriaceae	0.12±0.37	1.29±3.73	**0.036**
	ClostridialesvadinBB60group	0.96±2.81	0.15±1.09	**0.0031**
	Eubacteriaceae	0.008±0.03	0.21±0.85	0.38
	FamilyXIII_Unknown	3.55±13.36	0.85±2.11	0.20
	Lachnospiraceae	0.88±1.60	0.0003±0.001	**<0.0001**
	Ruminococcaceae	0.50±1.50	0.0003±0.001	0.20
	Leptotrichiaceae	8.01e-005±0.0003	0.0003±0.001	0.68
	Desulfovibrionaceae_Unknown	0.50±1.53	0.25±1.25	0.38
	Nesseriaceae	0.13±0.38	0.25±1.25	0.84
Genus	Actinomyces	0.01±0.05	1.23±2.75	**0.0023**
	Corynebacterium	0.017±0.074	1.41±0.62	**<0.0001**
	Lawsonella	0.00±0.00	0.056±0.40	**0.034**
	Rothia	0.09±0.40	0.52±2.13	0.2302
	Pseudopropionibacterium	0.06±0.20	0.99±5.50	**0.046**
	Elizabethkingia	7.19±22.79	1.22±5.49	**0.025**
	Nubsella	0.11±0.37	0.84±2.76	**0.089**
	Streptococus	0.23±0.80	0.91±2.79	**0.073**
	Pseudoramibacter	0.009±0.03	0.21±0.85	0.38
	Blautia	0.32±0.92	0.0008±0.002	0.19

	GCA_900066575	0.08±0.34	0.001±0.01	0.13
	Roseburia	0.11±0.27	0.0003±0.001	0.20
	Leptotrichia	8.02e-005±0.0003	0.0003±0.001	0.68
	Neisseria	0.05±0.22	0.44±1.62	**0.0099**
	Serratia	0.002±0.006	2.26±0.83	**0.0060**

Supplementary Table S3: Relative abundance (%) for taxonomic family and genus identified with significant differences in granuloma microbiota in the two groups among the high diversity samples. (data as mean ± SD. *p<0.05. **p<0.01. ****p<0.0001. unpaired Mann-Whitney test).

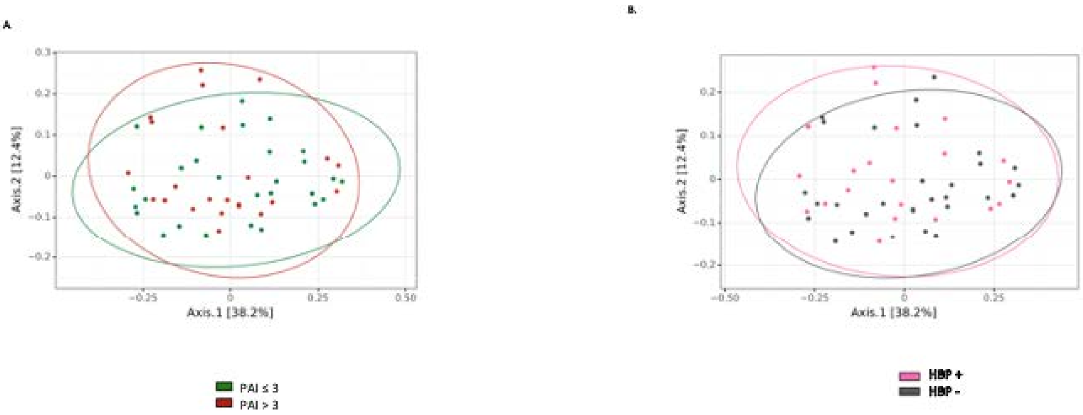

Supplementary Figure S1: Beta diversity
